# Olive Oil Quality Analysis and the Detection of Adulterations by GC‐MS With Cold EI

**DOI:** 10.1002/jms.70103

**Published:** 2026-08-14

**Authors:** Benny Neumark, Oneg Elkabets, Zadok Shabi, Aviv Amirav

**Affiliations:** ^1^ School of Chemistry Tel Aviv University Tel Aviv Israel; ^2^ Ado Analytical Consulting Rehovot Israel; ^3^ Aviv Analytical Hod Hasharon Israel

## Abstract

Olive oil is widely consumed for its nutritional and health benefits, yet its high market value makes it vulnerable to adulteration and mislabeling. Current analytical approaches for assessing olive oil quality and authenticity rely largely on indirect analyses, such as fatty acid methyl ester (FAME) profiles, peroxide values, or UV absorbance, and do not account for olive oil's triglycerides content. In this manuscript, we demonstrate the application of gas chromatography–mass spectrometry (GC‐MS) with cold electron ionization (Cold EI) for the direct analysis of olive oil based on its glyceride profiling. Cold EI employs a supersonic molecular beam interface with a contact‐free fly‐through ion source, providing vibrational cooling that enhances the abundance of molecular ions. This enables direct GC‐MS analysis of all glycerides and acids without derivatization, with total analysis time of 19 min. We analyzed multiple commercial olive oil brands, certified reference material, and intentionally adulterated samples containing canola and/or sunflower oil. The resulting mass chromatograms and triglyceride molecular ion patterns allowed clear discrimination between pure olive oil, adulterated oils, and degraded (oxidized) samples. Adulteration at the 30% level was detected through distinct hybrid triglyceride patterns, although aging effects were uniquely identified by increased diglyceride and free fatty acid signals. High sensitivity was demonstrated by the confident detection of olive oil at trace levels down to 100 pg on‐column using selective ion monitoring. These results establish GC‐MS with Cold EI as a rapid, sensitive, and highly informative technique for ensuring the authenticity, quality, and consumer safety of olive oil.

## 1 Introduction

With global annual consumption of about 3 billion liters, olive oil is widely used in healthy diets due to its rich content of monounsaturated fats, such as oleic acid, antioxidants, and anti‐inflammatory properties [1]. It offers significant health benefits, including improvements in cardiovascular health and potentially reduced cancer risk [2]. The International Olive Council sets various standards for olive oil producers and suggests quality testing regulations [3]. However, its high demand has led to the sale of counterfeit products that lack purity or freshness, posing a serious issue [4]. Current analytical methods often lack the selectivity needed to assess olive oil quality and authenticity based on its triglyceride (TAG) composition, underscoring the need for more advanced, reliable techniques to ensure olive oil quality and authenticity in the global market. Ideally, olive oil freshness and purity should be determined by analyzing its TAG content. Because TAG content cannot be analyzed directly, current analytical methods rely on profiling free fatty acids (e.g., fatty acid methyl esters [FAMEs]), peroxide value, and UV absorbance [4]. Current methods, such as high‐performance liquid chromatography (HPLC) with UV/Vis detection and GC with flame ionization detector (FID), exhibit poor selectivity for triglyceride analysis [5].

Gas chromatography–mass spectrometry (GC‐MS) has long been valued for its ability to separate and detect compounds in complex mixtures with excellent sensitivity and selectivity [6]. However, standard electron ionization (EI) often results in extensive molecular fragmentation, with the lack of the more selective high‐mass fragments and the molecular ion, the most characteristic ion. GC‐MS with standard EI is also limited to relatively low column flow rates (1–3 mL/min) and thus cannot analyze triglycerides at all (they do not elute) without hydrolysis and fatty acid derivatization, which is time consuming [7, 8].

Cold electron ionization (Cold EI) in GC‐MS is an advanced interface and ionization approach that substantially improves the performance of conventional EI‐based GC‐MS. The technique is based on a supersonic molecular beam (SMB) interface combined with a contact‐free fly‐through ion source. In this configuration, the GC column outlet is mixed with a makeup carrier gas, typically helium, that expands through a small pinhole or a shaped nozzle into a vacuum chamber, resulting in supersonic expansion. This supersonic expansion accelerates both the carrier gas and the analyte molecules to the same final velocity, resulting in highly uniform molecular motion and effective vibrational and rotational cooling of the analytes seeded in the SMB. The resulting cooling promotes enhanced molecular ion formation while retaining informative low‐mass fragment ions. Consequently, GC‐MS with Cold EI generates mass spectra that remain fully compatible with standard EI libraries, such as NIST, while providing increased molecular ion abundances and improved analytical robustness [9].

The main advantages of GC‐MS with Cold EI include efficient vibrational cooling of analyte molecules, which increases molecular ion signal intensity, and compatibility with high column flow rates (up to 100 mL/min). This capability enables rapid separations while operating at substantially lower elution temperatures. The contact‐free fly‐through EI ion source minimizes peak tailing and mitigates ion source contamination and degradation. Moreover, the unidirectional nature of the SMB enables effective jet separation and suppresses chemical background. Finally, the well‐defined kinetic energy of neutral analyte species enhances the effective filtration of the vacuum background, resulting in improved sensitivity and superior signal‐to‐noise performance.

By combining these features, Cold EI significantly boosts sensitivity, selectivity, and signal‐to‐noise ratios, making it a powerful tool for olive oil analysis. GC‐MS with Cold EI and its selected applications were recently reviewed in [10]. The principles and applications of GC‐MS with Cold EI are fully described in [11, 12, 13, 14, 15, 16, 17, 18, 19].

For olive oil analysis, GC‐MS with Cold EI provides detailed insights into its free fatty acids, vitamin E, and mono‐, di‐, and triglycerides. Cold EI produces rich triglyceride mass spectra with enhanced molecular ions, clearly indicating degrees of unsaturation. This facilitates accurate detection of fakes and freshness with minimal sample preparation and without hydrolysis, an integral step in standard olive oil analysis that can compromise results. These capabilities make GC‐MS with Cold EI a powerful tool for ensuring the authenticity and quality of olive oil.

## 2 Experimental

We used a 5977‐SMB GC‐MS with Cold EI system for this analysis. The system is based on an Agilent 7890B GC and 5977 MSD (Agilent Technologies, Santa Clara, CA, USA) integrated with the Aviv Analytical supersonic molecular beam (SMB) interface and a dual‐cage fly‐through ion source (Aviv Analytical LTD, Hod Hasharon, Israel). In this system, the GC column outlet is merged with helium makeup gas (~50 mL/min combined total column and makeup flow rate) just before a supersonic nozzle at the end of a heated and temperature‐controlled transfer line. Helium makeup gas can be mixed with perfluorotributylamine (PFTBA) through a valve for periodic system tuning and mass calibration. The sample compounds, seeded into the helium gas flow, are introduced into the SMB nozzle vacuum chamber through a 100‐μm‐diameter supersonic nozzle. This chamber is differentially pumped using a Varian Navigator 301 turbo molecular pump (Varian Inc., Torino, Italy) with a pumping speed of 250 L/s, maintaining a helium pressure of approximately 6 × 10^−3^ mbar. The supersonic expansion cools the sample molecules, and a 0.8‐mm skimmer skims the expanded jet before it is collimated into a second, differentially pumped vacuum chamber. The SMB is formed and directed into a fly‐through dual‐cage EI ion source. The second vacuum chamber and the ion source are maintained by the Agilent 5977 system “Performance” turbo molecular pump (Pfeiffer, 250 L/s). Ionization is achieved using 70 eV electrons with 6 mA emission current. Notably, the Cold EI ion source was operated for over 12 years without requiring maintenance and has maintained consistent performance. An ion lens system focuses and deflects the generated ions by 90° using an ion mirror, which is separately heated to prevent contamination of the mass analyzer. The ions are then analyzed by the Agilent 5977 MS and detected using the triple‐axis ion detector. Data acquisition and processing were conducted using Agilent ChemStation and MassHunter software.

GC separation was performed on a 15‐m column (0.32 mm I.D., 0.1 μm DB1‐HT film) with a column flow program starting at 2 mL/min for 12 min, followed by a 10 mL/min/min increase up to a rate of 32 mL/min with a hold time of 3 min. The GC oven was programmed from 50°C (0.5 min hold) and ramped to 280°C at 20°C/min and then ramped again at 40°C/min to 350°C (5.25 min hold), with a total run time of 19 min. The sample injection used an Agilent Multi‐Mode Inlet (MMI) set to 300°C, with an 800°C/min ramp to 350°C (5 min hold). The MMI operated in split mode (split ratio 1:9). Seven different local Israeli olive oil brands, two Italian brands, one Spanish brand, and a certified reference material (CRM) were analyzed. All the olive oil brands, both local and imported, were purchased at a local supermarket. The CRM was purchased from Merck Corporation (Darmstadt, DE), P/N 1478254.

## 3 Results

**FIGURE 1:**
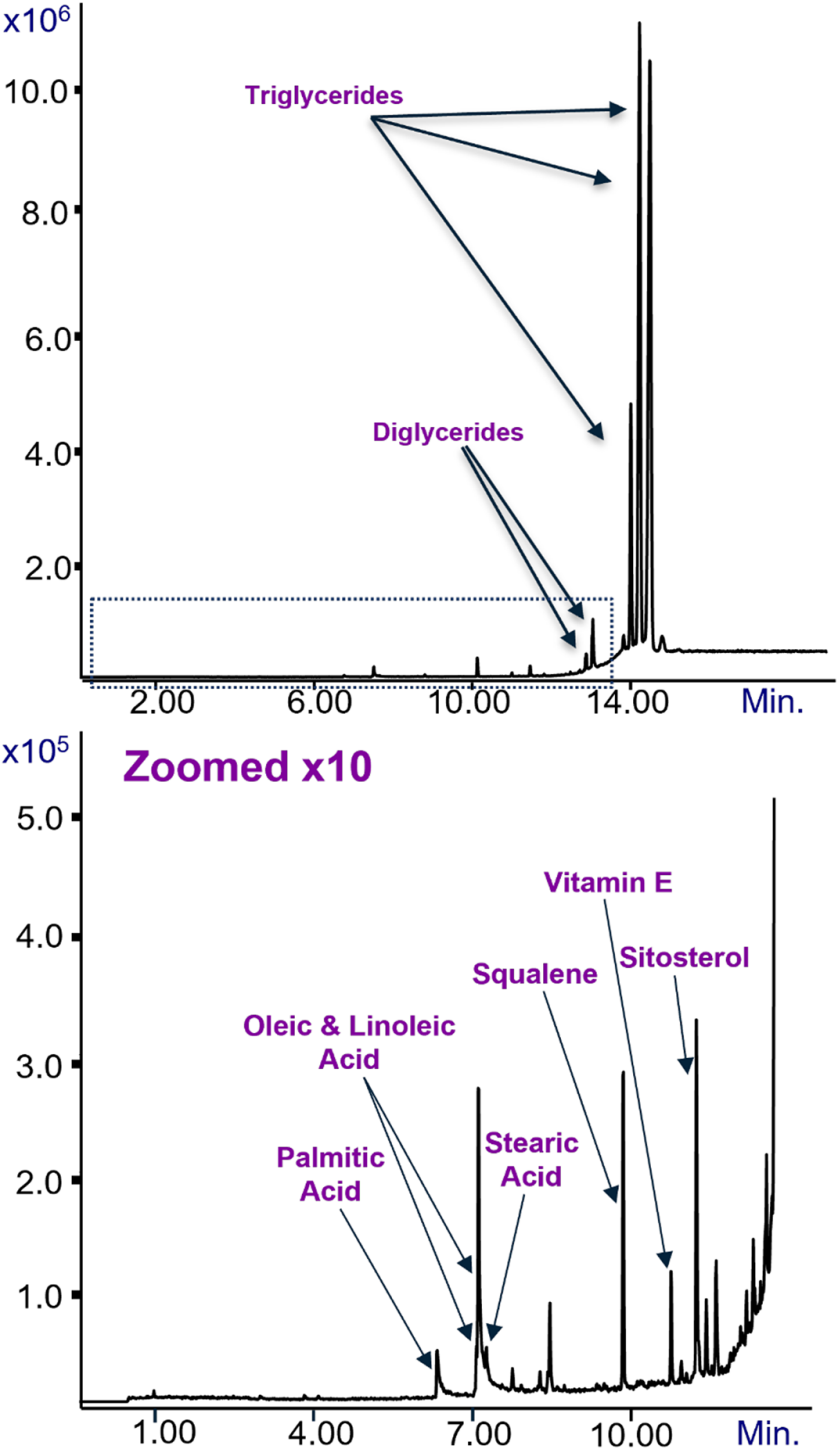
On top, a mass chromatogram obtained from the analysis of pure olive oil diluted to 5000 ppm in hexane. The arrows indicate the peaks for diglycerides and triglycerides. At the bottom, a 10× zoom‐in on the first 14 min of the analysis (marked with a rectangle) shows a wide range of additional compounds detected.

Figure 1 shows the results from the analysis of 5000 ppm pure olive oil diluted in hexane. We note that the analysis time is only 19 min. The mass chromatogram is rich in information, showing a wide range of compounds, including palmitic acid, oleic and linoleic acids, stearic acid, squalene, vitamin E, and sitosterol, as well as diglycerides (DAG) and TAG. The GC‐MS analysis using Cold EI is much more informative than with standard EI or other alternatives, as it provides additional information beyond that mentioned above, including the free fatty acids, DAG and TAG identities, along with their distributions and intensity ratios. Furthermore, we found that as shown in Figure 1, olive oil includes very little or no volatile organic compounds, and thus, if it smells, it could be due to TAG degradation and possibly the addition of terpene compounds and alike.

**FIGURE 2:**
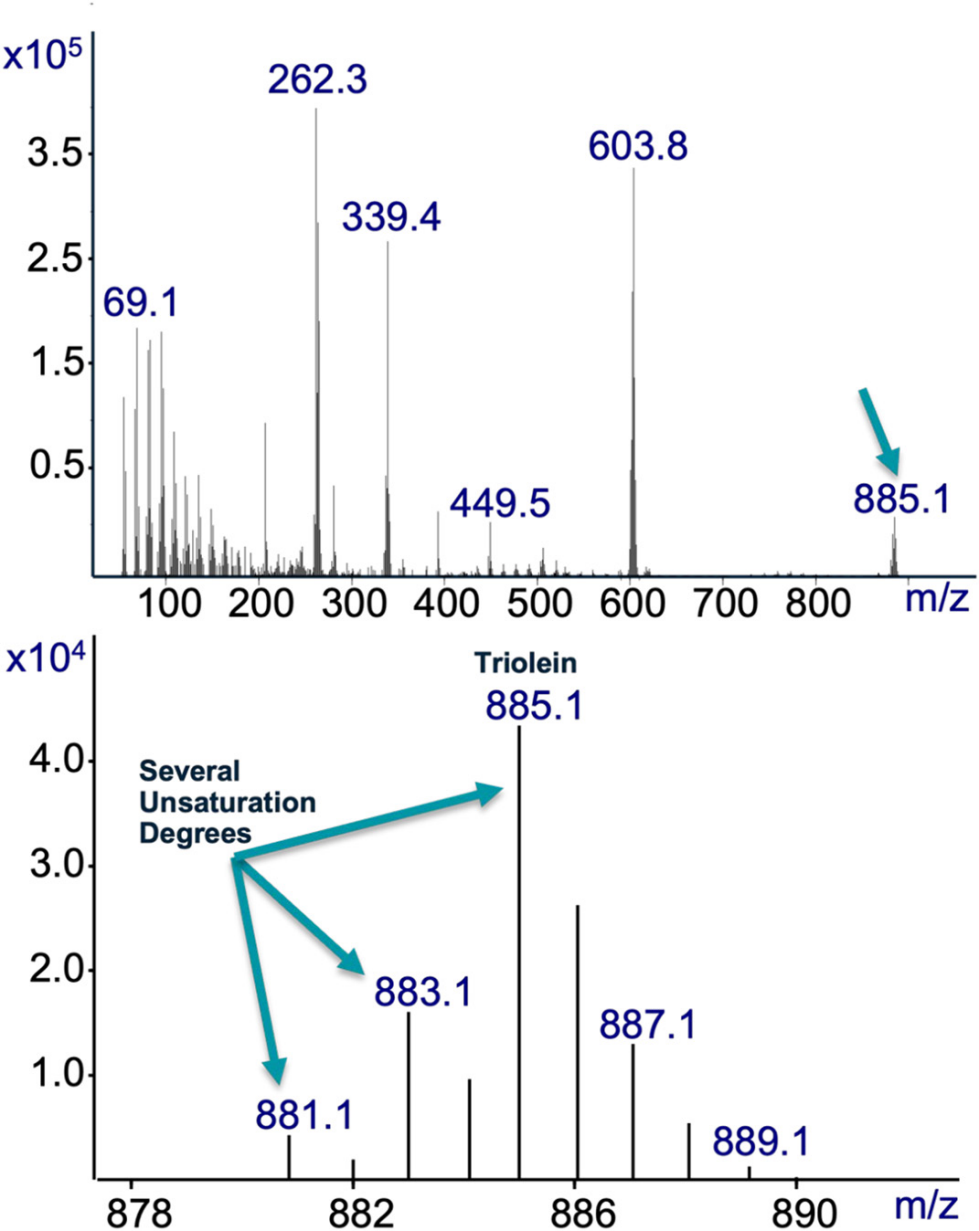
On top, the Cold EI mass spectrum of triolein. We note the molecular ion at m/z = 885.1. On the bottom, a zoom‐in on the molecular ions, showing different degrees of unsaturation.

Figure 2 shows the mass spectrum of triolein mass chromatogram peak. Because GC‐MS with Cold EI extends the range of molecules amenable to GC‐MS analysis, the mass spectrum shows an enhanced molecular ion at m/z = 885 for this heavy compound. Zooming in on the molecular ion reveals additional information, showing several degrees of unsaturation, including m/z = 883 and 881, that are likely due to having one or two linoleic acids.

**FIGURE 3:**
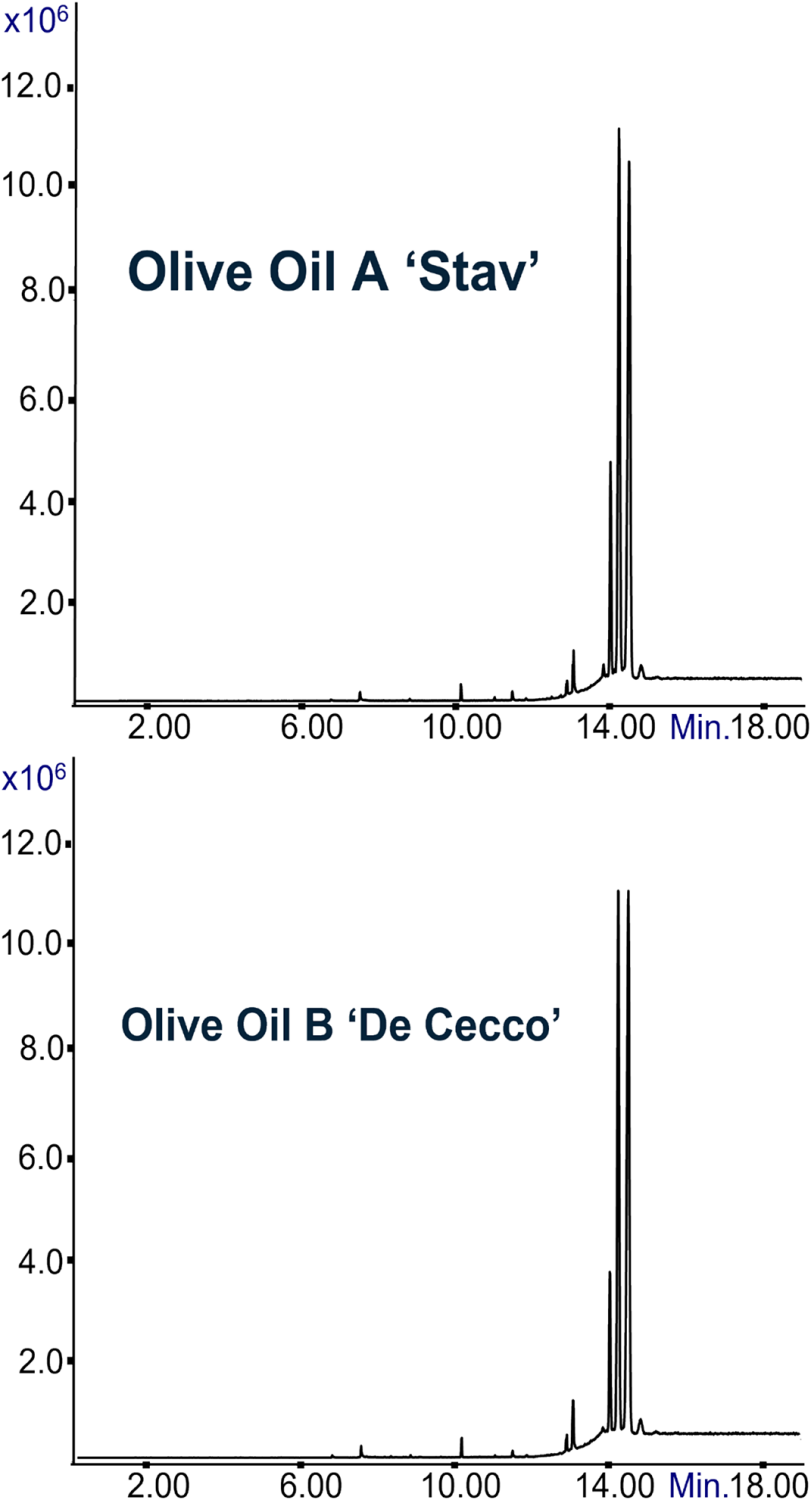
On top, the mass chromatogram of the analysis of 5000 ppm in hexane “Stav” Israeli olive oil. On the bottom, the mass chromatogram of the analysis of 5000 ppm in hexane “De Cecco” Italian olive oil. We note that the two mass chromatograms are nearly identical.

We assumed that local and imported brands would exhibit distinct mass chromatograms due to differences in environmental and climatic conditions. However, to our surprise, the results were highly reproducible, with no noticeable differences across brands. Figure 3 shows the mass chromatogram from the analysis of 5000 ppm pure olive oil of the Israeli brand “Stav” and the Italian brand “De Cecco.”

**FIGURE 4:**
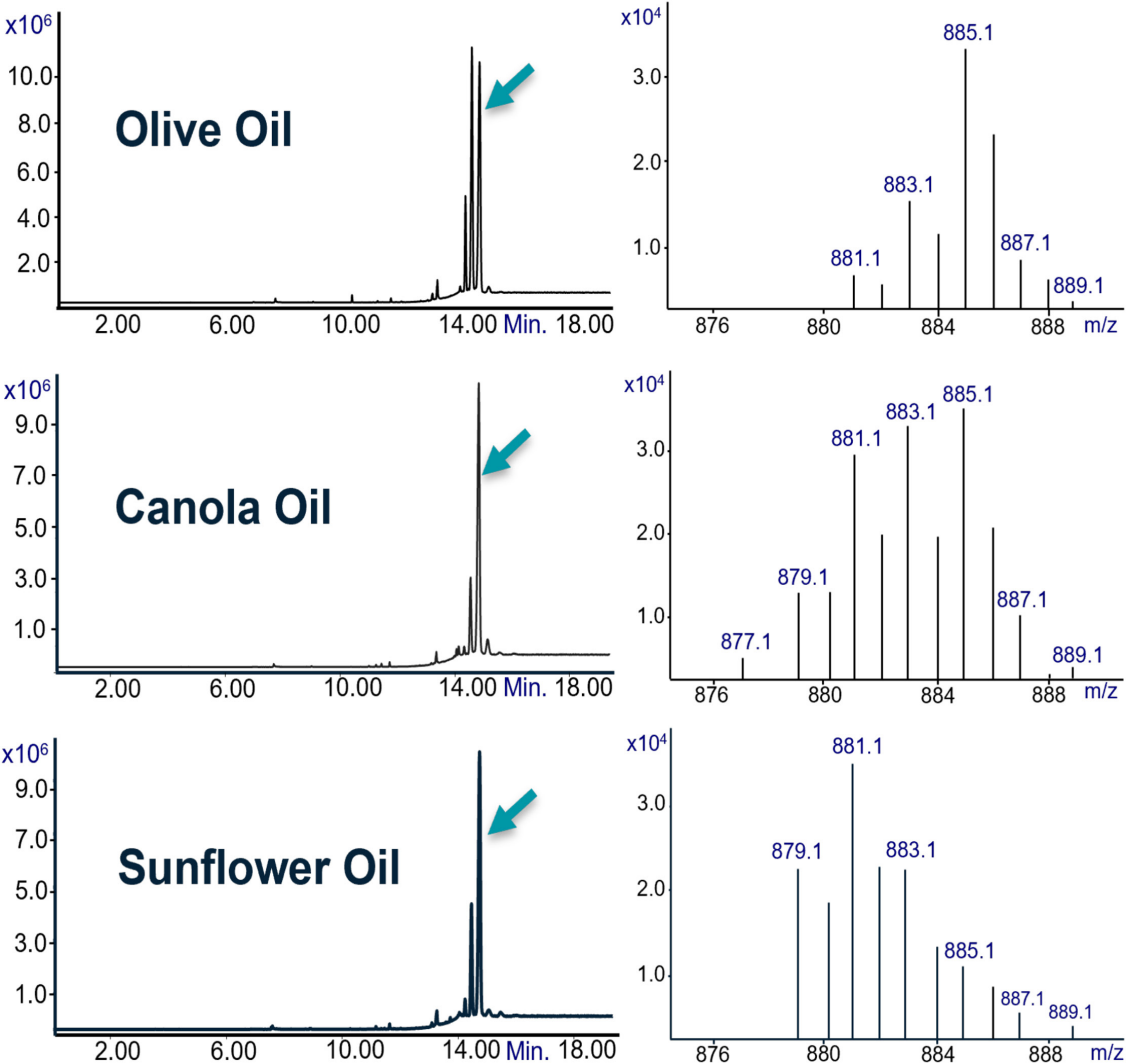
On the left, the mass chromatogram of the analysis of 5000 ppm in hexane, olive oil, canola oil, and sunflower oil. On the right, a zoom‐in of the molecular ions of the peaks that are indicated by the arrows. This comparison shows that GC‐MS with Cold EI can distinguish different oil types based on their triglyceride content, as evidenced by the mass chromatogram. Zooming in on the molecular ion of the indicated triglyceride further supports that conclusion.

**FIGURE 5:**
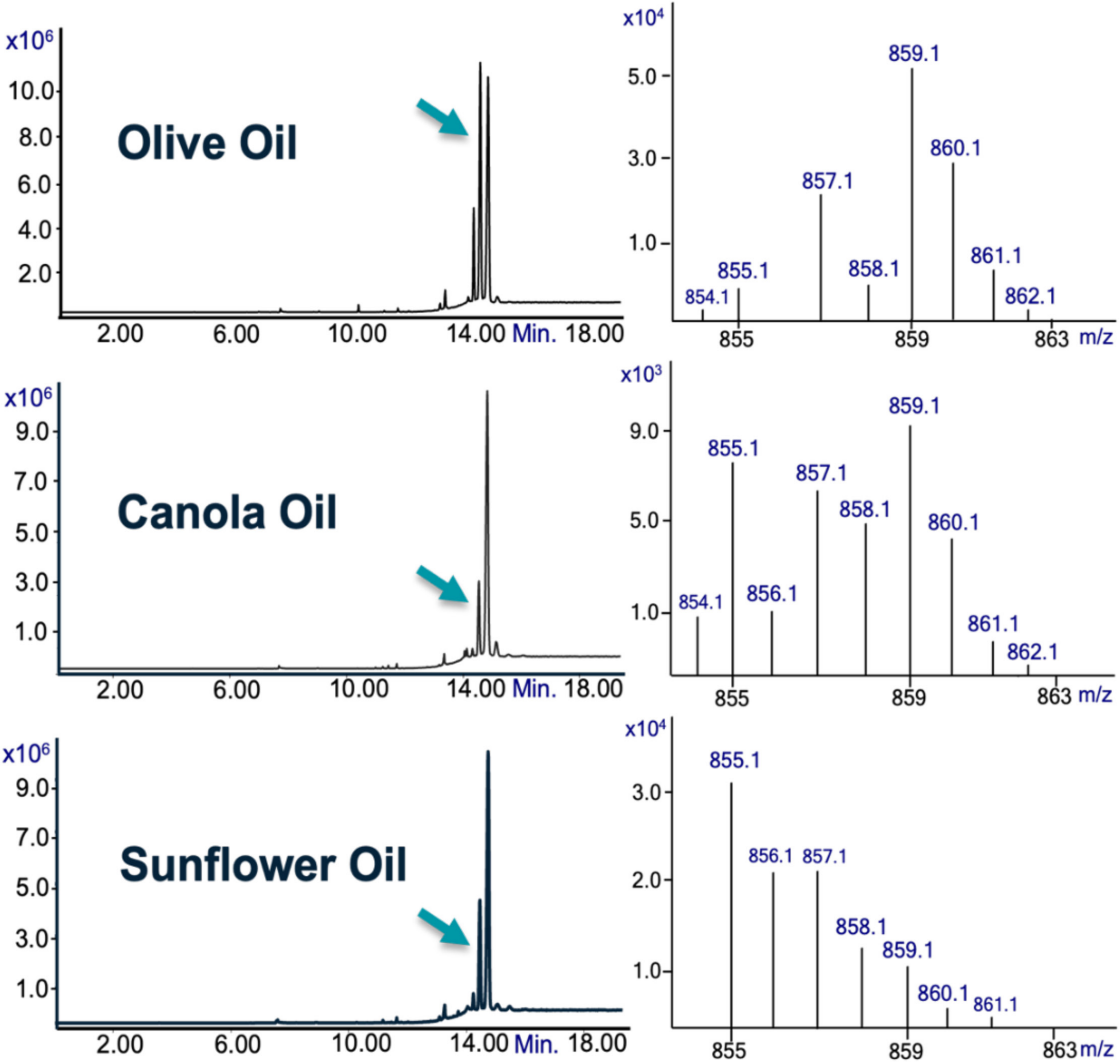
On the left, the mass chromatograms of the analysis of 5000 ppm in hexane for olive oil, canola oil, and sunflower oil. On the right, a zoom‐in of the molecular ion of the peaks indicated by the arrows. This comparison shows that GC‐MS with Cold EI can distinguish different oil types based on their triglyceride composition, with more pronounced differences observed for this triglyceride compared to triolein (Figure 4). Zooming in on the molecular ion of the indicated triglyceride further emphasizes these distinctions and highlights the unique spectral pattern of each oil.

**FIGURE 6:**
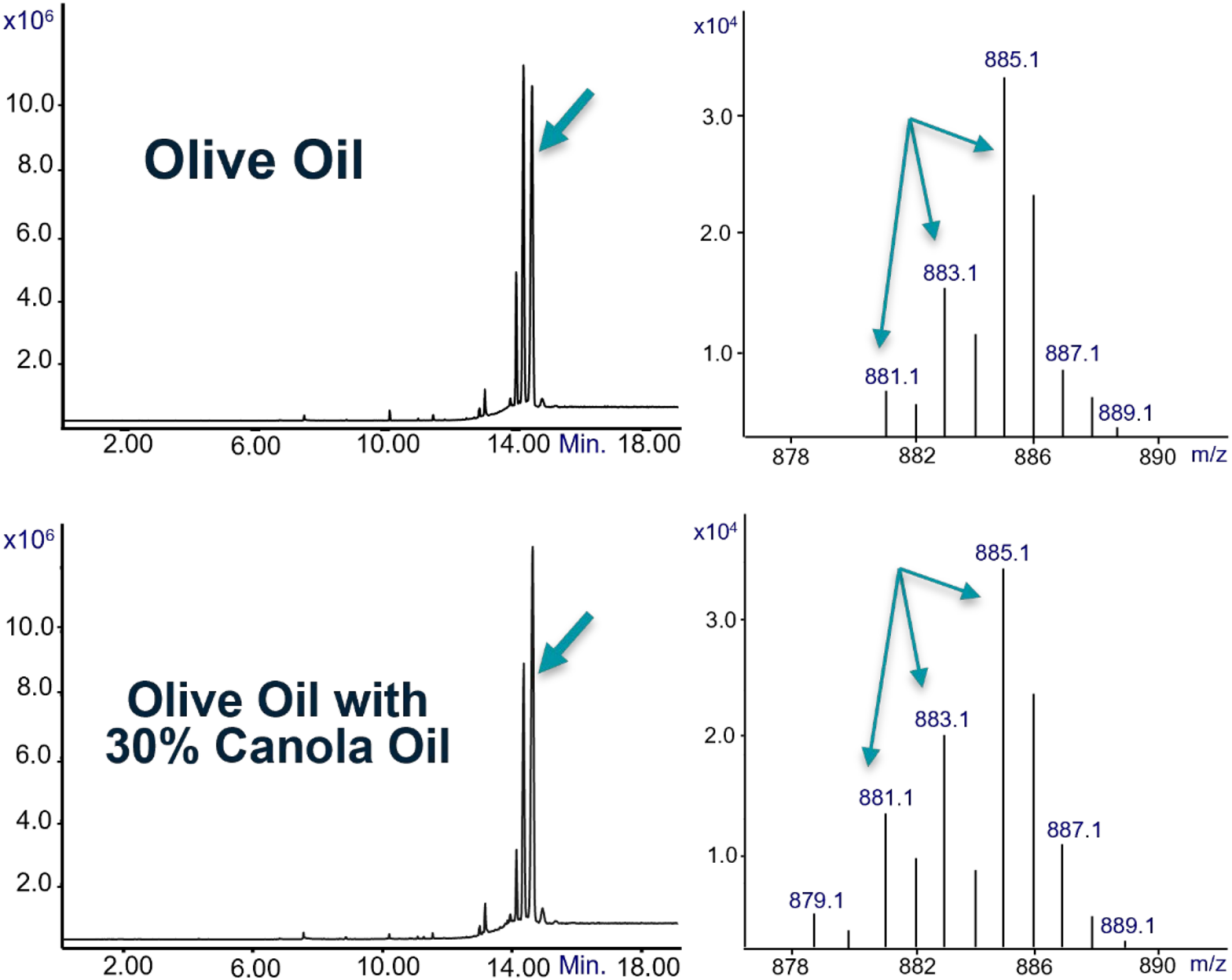
On the left, the mass chromatograms generated from the analysis of 5000 ppm in hexane, olive oil (upper) and olive oil spiked with 30% canola oil (bottom). On the right, a zoom‐in of the molecular ions indicated by the arrows. Zooming in on the molecular ions of the indicated triglyceride reveals a unique hybrid pattern, immediately suggesting adulterated olive oil.

**FIGURE 7:**
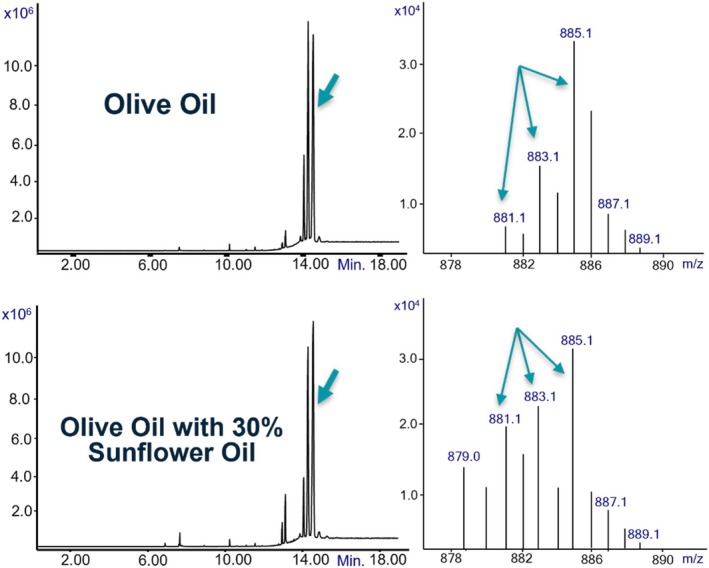
On the left, the mass chromatograms of the analysis of 5000 ppm in hexane of olive oil (upper) and olive oil spiked with 30% sunflower oil. On the right, a zoom‐in of the molecular ions of the peaks indicated by the arrows. As shown in Figure 6, for olive oil spiked with sunflower oil, zooming in on the molecular ion of the indicated triglyceride reveals a unique hybrid pattern, immediately suggesting that the oil is adulterated.

Based on our research, we found that the most common method of producing counterfeit olive oil is to mix it with a cheaper oil. The two most commonly used, less expensive oils for this are canola and sunflower oils [20]. These two oil types do not affect the taste or smell of olive oil, making them excellent candidates for oil adulteration [21]. Figure 4 shows the results of the analysis of 5000 ppm olive oil, canola oil, and sunflower oil in hexane. The mass chromatograms of canola oil and sunflower oil are clearly different from that of pure olive oil. In addition, the molecular ions of the TAG indicated by the arrow show a distinct pattern for each oil type with olive oil having the lowest degree of unsaturation (purer triolein). This is due to differences in the ratios of unsaturated triglycerides across kinds [22]. Note that the main triglyceride peak is relatively broad due to co‐elution of several isomers and mixtures of different triglycerides. This comparison clearly shows that pure olive oil can be easily identified by its triglyceride content and immediately detected from a GC‐MS with a Cold EI mass chromatogram. Moreover, the molecular ions of the triglyceride peak, indicated by the arrow, provide additional confirmation based on its unique mass spectrum. Figure 5 presents a comparison of the three oil types for an additional triglyceride. Based on its mass spectrum, we assume that this is a mixed TAG containing palmitic, oleic, and linoleic fatty acids. Whereas the variations observed in the mass chromatograms for triolein (Figure 4) are relatively subtle, the differences in the chromatographic peak indicated by the arrow in Figure 5 are more pronounced. A closer examination of the molecular ions further highlights these distinctions. As shown in the figure, the mass spectrum of this TAG exhibits a unique pattern for each oil, providing an additional distinctive basis for differentiation. Based on our research, we found that adulterating olive oil with cheaper oil becomes cost‐effective when at least 30% of the cheaper oil is used. Figures 6 and 7 present the results of the analysis of 5000 ppm hexane in olive oil spiked with 30% canola oil (Figure 6) and sunflower oil (Figure 7). In the triglyceride region, it is immediately clear in both cases that this is not pure olive oil. Once again, a zoom‐in of the molecular ions of the TAG, indicated by the arrow, reveals a hybrid pattern and supports the conclusion that this is not pure olive oil. Clearly, both canola and sunflower oils contain more TAG with one or two linoleic acids than olive oil, and the sunflower oil's molecular ion with the most abundant peak is with two linoleic acids and one oleic acid (m/z = 881.1). In addition, olive oil is also characterized by a larger triglyceride peak containing one palmitic acid.

**FIGURE 8:**
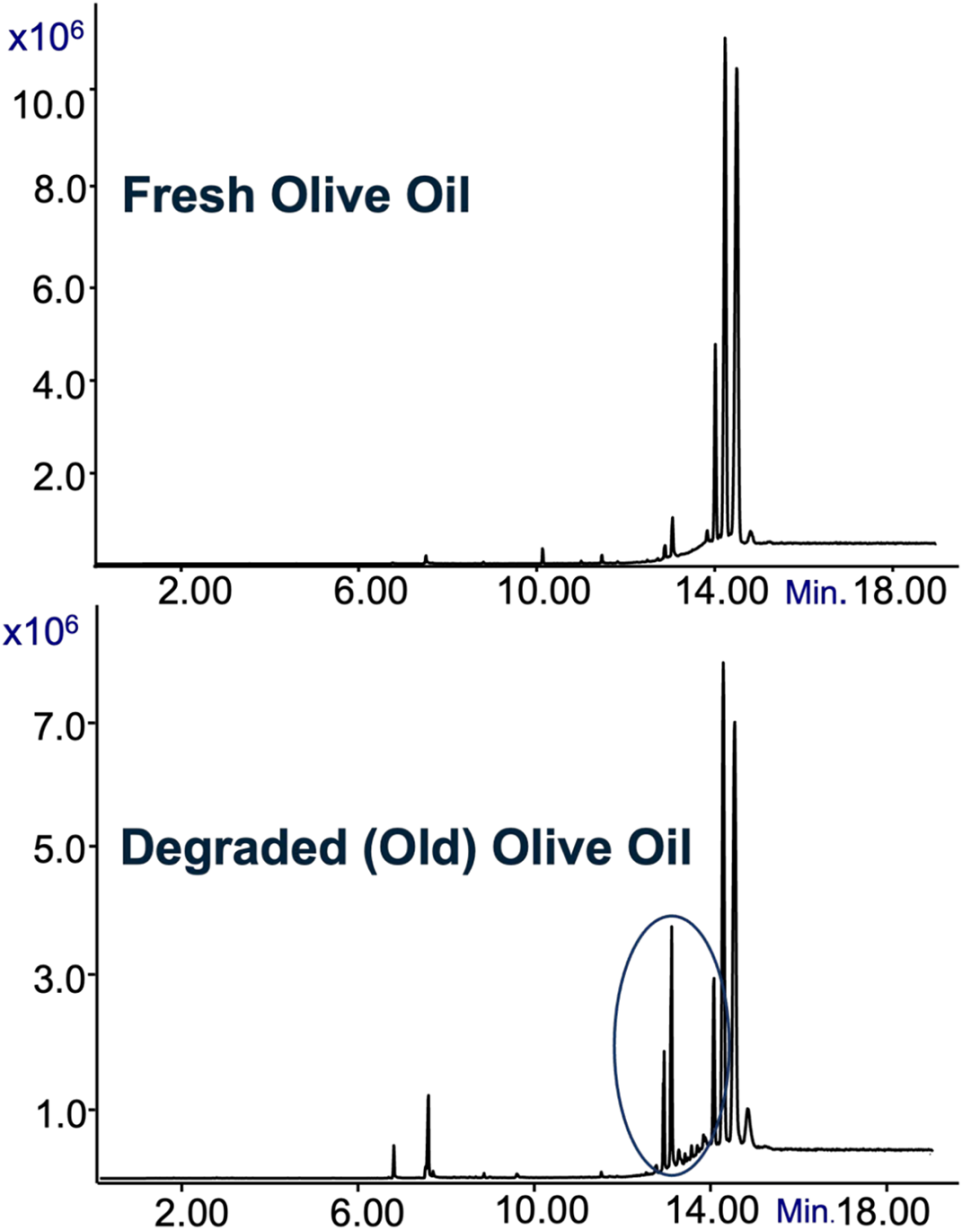
On the top, the mass chromatogram of the analysis of 5000 ppm in hexane, fresh olive oil. On the bottom, the mass chromatogram of the analysis of 5000 ppm in hexane, old, degraded olive oil. As shown in the bottom trace (circled), the diglyceride region and free fatty acids are much more abundant (higher) in the old olive oil. We note that this is probably due to the oxidation and/or degradation of triglycerides over time.

Another common method of adulterating olive oil is mixing pure, fresh olive oil with old, degraded olive oil. Figure 8 compares the analysis of fresh olive oil (5000 ppm diluted with hexane) and of degraded (old) ~5 years old, olive oil (5000 ppm diluted in hexane). The mass chromatogram region corresponding to TAG and DAG clearly indicates that this is not fresh olive oil. This is evident from the highly increased levels of free fatty acids and diglycerides. We assume that this is due to the decomposition of the triglycerides over time. We note that this feature of olive oil freshness quality detection is unique to Cold EI and not possible with GC‐MS with standard EI that requires hydrolysis of the TAG and fatty acids conversion into FAMEs and thus loses any separate information on free fatty acids and DAG concentrations.

**FIGURE 9:**
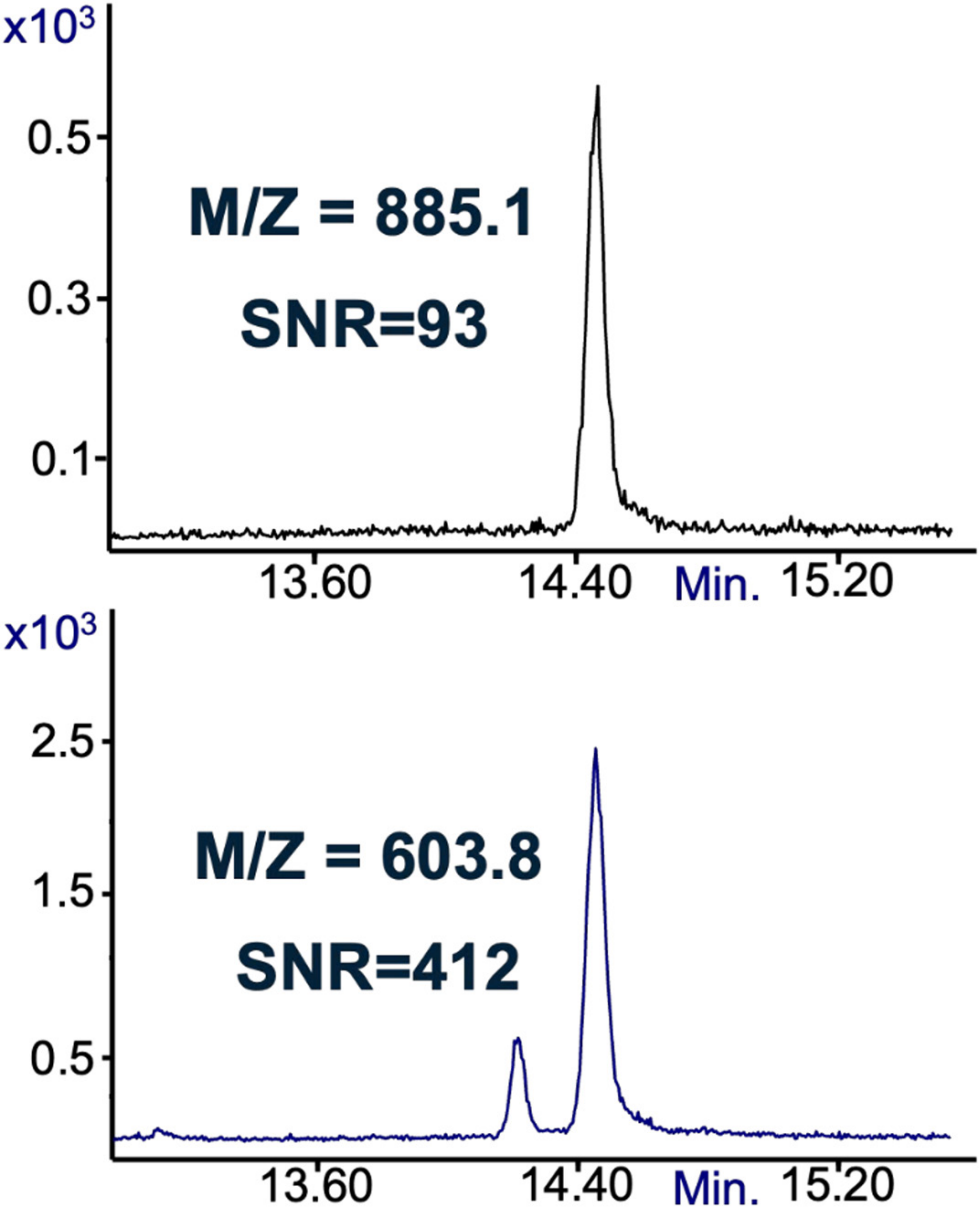
The results from the analysis of 100 pg on‐column amount of fresh olive oil diluted in hexane in SIM mode. The results show that GC‐MS with Cold EI readily detects trace levels of triolein. The signal‐to‐noise ratios (SNR) are in peak‐to‐peak units.

We also aimed to assess the sensitivity of GC‐MS with Cold EI for trace levels of olive oil. Figure 9 shows the results of analyzing 1 ppm olive oil diluted in hexane by GC‐MS with Cold EI, with a split injection ratio of 9:1, resulting in an on‐column amount of 100 pg. The analysis was performed in selective ion monitoring (SIM) mode for m/z = 885.1 (the most abundant ion of triolein) and m/z 603.8 that is an abundant fragment ion of triolein as shown in Figure 2. The figure shows that trace levels of triolein can be detected with great confidence and selectivity using GC‐MS with Cold EI with a calculated limit of detection (LOD) of about one picogram based on the peak‐to‐peak signal‐to‐noise ratio as shown in Figure 9.

## 4 Conclusions

In this research, we demonstrated the application of GC‐MS with Cold EI for the direct analysis of olive oil based on its full content and TAG composition. The results show that GC‐MS with Cold EI is a highly informative and efficient method for evaluating the authenticity, quality, and freshness of olive oil. Conventional analytical techniques for olive oil authentication rely largely on indirect measurements, such as FAME profiles, peroxide values, or spectrophotometric analyses. Although useful, these approaches do not directly analyze the free fatty acid and TAG composition of olive oil, which is the most characteristic chemical feature of edible oils. By enabling direct GC‐MS analysis of mono‐, di‐, and triglycerides with a minimal step of dilution as sample preparation, GC‐MS with Cold EI yields richer, more diagnostically informative results for olive oil analysis. The experimental results demonstrate several important analytical advantages of GC‐MS with Cold EI, such as reproducible mass chromatograms that include free fatty acids, sterols, vitamins, diglycerides, and triglycerides in a single analysis. The enhanced molecular ions achieved through vibrational cooling in SMB enable clear identification of high‐mass TAG that are typically difficult to observe using standard electron ionization. This capability provides a direct molecular fingerprint of olive oil that reflects its TAG composition. Second, the results show that pure olive oil from different geographical sources produces highly similar chromatographic and mass spectral patterns. This reproducibility confirms that the TAG profile of genuine olive oil is highly characteristic and can serve as a reliable reference signature. Vegetable oils, such as canola and sunflower oils, also exhibit distinct yet different triglyceride molecular ion patterns. We showed that the molecular ion of triolein, the most abundant triglyceride in all three oil types analyzed in this study, exhibits only subtle differences in the mass chromatogram. However, the mass spectra of its molecular ions display a distinctive pattern for each oil type with different percentages of linoleic acid. In addition, the second most abundant triglyceride shows even more pronounced differences in both the mass chromatograms and the molecular ions spectral patterns. These differences allow rapid discrimination between oil types based on their molecular ion distributions and mass chromatogram profiles. The results also demonstrated strong performance in detecting olive oil adulteration. When olive oil was intentionally mixed with cheaper vegetable oils, the resulting chromatograms clearly showed hybrid triglyceride patterns that deviated from those of pure olive oil. Adulteration at the 30% level was readily detected, confirming that GC‐MS with Cold EI can effectively identify economically motivated adulteration practices. Importantly, these differences are evident directly in both the chromatographic and molecular ion patterns, enabling straightforward interpretation without complex chemometric processing. In addition to detecting mixing with other oils, the technique also uniquely identified degraded olive oil. Aged samples exhibited elevated free fatty acid and diglyceride signals compared with fresh olive oil. These changes likely reflect the triglyceride hydrolysis degradation process that occurs during prolonged storage. Therefore, GC‐MS with Cold EI can uniquely provide useful information not only on authenticity but also on the freshness and chemical stability of olive oil products, which cannot be achieved with standard EI using FAME analysis. Another key advantage of the method is its analytical speed and simplicity. The entire analysis requires only 19 min and does not require derivatization or extensive sample preparation. In comparison, traditional FAME‐based analyses typically require hydrolysis and derivatization steps followed by significantly longer chromatographic runs. Eliminating these preparation steps reduces analysis time, minimizes potential sources of error, and simplifies routine laboratory workflows. The sensitivity of GC‐MS with Cold EI further enhances its analytical value. The successful detection of olive oil at trace levels, down to approximately 100 pg on‐column, demonstrates the technique's high signal‐to‐noise performance. Such sensitivity may be valuable in applications requiring trace detection, including contamination studies, forensic investigations, or the analysis of minute food residues. Taken together, these results show that GC‐MS with Cold EI offers a powerful and comprehensive approach for olive oil analysis. By directly characterizing glyceride composition while maintaining compatibility with standard EI mass spectral libraries, the technique combines the strengths of traditional GC‐MS with significantly enhanced molecular information. This capability enables rapid detection of adulteration, assessment of oil freshness, and confident identification of an olive oil sample. Given the global economic importance of olive oil (over $20 Billions/year world market) and the ongoing problem of food fraud, analytical methods that provide reliable, direct chemical characterization are critically important. GC‐MS with Cold EI has the potential to become an important tool for regulatory laboratories, food quality control facilities, and research institutions working to ensure the authenticity and safety of olive oil products. In addition, the method may be extended to other edible oils and lipid‐rich food products where TAG composition plays a key role in authenticity and quality assessment. Overall, the results presented here demonstrate that GC‐MS with Cold EI is a well‐suited analytical technology for olive oil characterization. Its ability to directly analyze glycerides with high sensitivity, minimal sample preparation, and rapid analysis time provides a significant advancement over traditional approaches. As a result, GC‐MS with Cold EI offers a promising pathway toward more reliable and informative methods for ensuring olive oil authenticity, quality, and consumer protection.

## Funding

This work was supported by Israel Science Foundation (312/22).

## Data Availability

The data that support the findings of this study are available from the corresponding author upon reasonable request.
